# Photobiomodulation Inhibits Ischemia-Induced Brain Endothelial Senescence via Endothelial Nitric Oxide Synthase

**DOI:** 10.3390/antiox13060633

**Published:** 2024-05-23

**Authors:** Yu Feng, Zhihai Huang, Xiaohui Ma, Xuemei Zong, Vesna Tesic, Baojin Ding, Celeste Yin-Chieh Wu, Reggie Hui-Chao Lee, Quanguang Zhang

**Affiliations:** 1 Institute for Cerebrovascular and Neuroregeneration Research, Shreveport, LA 71103, USA; yu.feng@lsuhs.edu (Y.F.); zhihai.huang@lsuhs.edu (Z.H.); xuemei.zong@lsuhs.edu (X.Z.); vesna.tesic@lsuhs.edu (V.T.); yinchieh.wu@lsuhs.edu (C.Y.-C.W.); 2 Department of Neurology, Louisiana State University Health, Shreveport, LA 71103, USA; xiaohui.ma@lsuhs.edu; 3 Department of Biochemistry & Molecular Biology, Louisiana State University Health, Shreveport, LA 71103, USA; baojin.ding@lsuhs.edu

**Keywords:** angiogenesis, cerebrovascular senescence, endothelial nitric oxide synthase, photobiomodulation, stroke

## Abstract

Recent research suggests that photobiomodulation therapy (PBMT) positively impacts the vascular function associated with various cerebrovascular diseases. Nevertheless, the specific mechanisms by which PBMT improves vascular function remain ambiguous. Since endothelial nitric oxide synthase (eNOS) is crucial in regulating vascular function following cerebral ischemia, we investigated whether eNOS is a key element controlling cerebrovascular function and the senescence of vascular endothelial cells following PBMT treatment. Both rat photothrombotic (PT) stroke and in vitro oxygen–glucose deprivation (OGD)-induced vascular endothelial injury models were utilized. We demonstrated that treatment with PBMT (808 nm, 350 mW/cm^2^, 2 min/day) for 7 days significantly reduced PT-stroke-induced vascular permeability. Additionally, PBMT inhibited the levels of endothelial senescence markers (senescence green and p21) and antiangiogenic factor (endostatin), while increasing the phospho-eNOS (Ser1177) in the peri-infarct region following PT stroke. In vitro study further indicated that OGD increased p21, endostatin, and DNA damage (γH2AX) levels in the brain endothelial cell line, but they were reversed by PBMT. Intriguingly, the beneficial effects of PBMT were attenuated by a NOS inhibitor. In summary, these findings provide novel insights into the role of eNOS in PBMT-mediated protection against cerebrovascular senescence and endothelial dysfunction following ischemia. The use of PBMT as a therapeutic is a promising strategy to improve endothelial function in cerebrovascular disease.

## 1. Introduction

Stroke is characterized by a sudden neurological event resulting from inadequate blood flow to the brain’s blood vessels. Ischemic stroke, which accounts for 85% of all strokes [1], has a high risk of long-term recurrence and disability. One of the major hallmarks following ischemic stroke is the inherent endothelial senescence, which leads to blood–brain barrier (BBB) dysfunction. Therefore, restoring endothelial function within the ischemic region offers immense benefits in the treatment against stroke. As such, endothelial nitric oxide synthase (eNOS), which catalyzes the conversion of L-arginine and oxygen to nitric oxide (NO), is a key indicator of endothelial and vascular function [2,3]. In fact, the impairment of eNOS activity due to ischemic stroke and aging has been implicated in numerous cellular mechanisms involving sphingomyelinase- and ceramide-activated phosphatase 2A or AKT pathways, leading to neuronal injury [4,5]. For example, deletion of eNOS in mice subject to ischemic stroke develops a larger infarct size as compared to the wild-type variant, while activation of eNOS confers protection against stroke by inhibiting endothelial dysfunction. These discoveries highlight the significance of eNOS as a promising therapeutic target in the treatment of stroke [6,7,8].

The use of photobiomodulation therapy (PBMT) as an alternative treatment for various neurodegeneration diseases (AD, ischemic stroke, brain injury, and Parkinson’s disease) has received increasing attention in the past few years [9,10,11,12,13,14]. Transcranial PBM, the process of delivering light photons through the skull to benefit from their modifying effect, has been widely used in preclinical experiments and clinical trials for treating brain diseases [15,16]. We previously discovered that post-treatment with PBMT in a photothrombotic (PT) stroke animal model can alleviate behavioral deficits and confer cerebrovascular protection. This is attributed to PBMT’s unique role in promoting neurogenesis, preserving blood–brain barrier (BBB) integrity, facilitating angiogenesis, and mitigating vascular permeability [9,10,17,18,19]. Although the underlying mechanism remains controversial, PBMT has been shown to enhance NO generation in vitro [20]. This discovery naturally led us to the hypothesis that the beneficial effects of PBMT on vascular function may be mediated through eNOS. We thus investigated whether PBMT can preserve vascular function by inhibiting vascular senescence in the PT stroke animal model. More importantly, this study seeks to validate the role of eNOS in mediating the beneficial effects of PBMT in the mouse brain microvascular endothelial cell line.

## 2. Materials and Methods

### 2.1. Animals and Study Design

Animal use and experimental protocols were approved by the Institutional Animal Care and Use Committee of the Louisiana State University (LSU) Health Sciences Center, Shreveport, LA, USA. All experimental procedures followed the guidelines set by the Institutes for the Humane Care and Use of Laboratory Animals and were in accordance with the ARRIVE guidelines. Male Sprague–Dawley rats (250–300 g) were utilized in this study. The rats were housed in pairs (2 rats/cage from the same litter) in a temperature- and light-controlled environment (maintained at 23 °C with a 12-h light/dark cycle) with ad libitum access to food and water. Random assignment of rats into three groups was conducted: (a) control group (sham group), (b) experimental group with PT stroke (PT group), and (c) experimental group with PT stroke receiving PBMT (PT + PBMT group). Experimenters were blinded to group assignments.

### 2.2. PT Stroke Injury

The PT model was to produce a more reproducible cortical infarct without craniotomy in rats. The PT stroke model was established following the methodology outlined in our previous study [21]. Briefly, rats were initially anesthetized with isoflurane and securely placed in a stereotactic frame. Rose Bengal dye (0.1 mg/g) was then administered intraperitoneally over 5 min before exposing the skull. After delicately removing the periosteum, the skull underwent exposure to a 6 mm diameter cold white light beam for a duration of 15 min. This light was meticulously positioned 1.8 mm anterior to the bregma and 2.5 mm lateral from the midline. Throughout the surgical procedure, animal body temperature was meticulously maintained at 37 ± 0.5 °C by placing them on a heating pad. After the procedure, wound closure was performed using wound clips. Carprofen (5 mg/kg, subcutaneous injection) was provided at surgery and continued twice daily for 48–72 h post-surgery. Animals were monitored closely twice a day for 3 days following the surgery.

### 2.3. PBM Treatment

**Figure 1:**
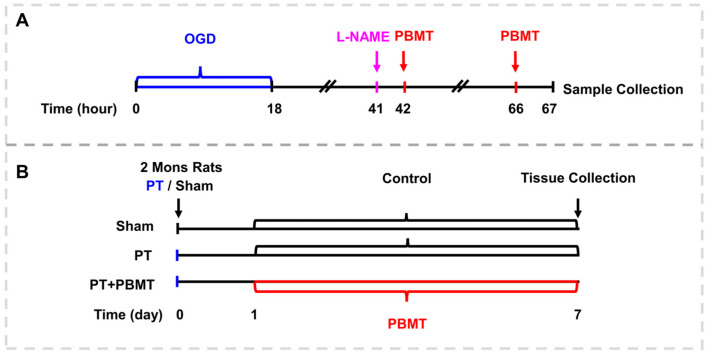
Schematic diagram. (**A**) The experimental procedure for culturing the bEnd.3 cells. (**B**) Experimental timeline for animal experiments.

The detailed procedure for PBMT is illustrated in Figure 1. As reported in our previous study [17], using a diode IR laser system (808 M100, Dragon Lasers Co., Ltd., Changchun, China), 2-minute daily laser irradiations (808 nm, 350 mW/cm^2^ on the scalp) were applied on the scalp area underlying the infarct injury from day 1 to day 7. Rats were gently restrained with a clear cone (DCL-120, Braintree Scientific, Braintree, MA, USA) during the course of therapy. The laser spot measured approximately 1.5 cm^2^, and the distance measured between the laser point and the rat scalp was 35 cm. In order to perform PBMT in vitro, bEnd.3 cells were subjected to a 25 mW/cm^2^ irradiance for two minutes (fluence: 3 J/cm^2^, laser spot: 2 cm^2^/well for 24-well plates, 9.6 cm^2^/well for 6-well plates), starting 24 h and 48 h after OGD.

### 2.4. Brain Perfusion and Tissue Preparation

The rats were put under profound anesthesia caused by isoflurane and then transcardially perfused with ice-cold PBS, as previously reported in our study [11]. Brain tissues were cryopreserved in a 30% sucrose solution after being post-fixed for an overnight period in 4% paraformaldehyde (PFA). Following this, coronal sections (25 μm in thickness) from the ischemic infarct cortex were obtained using a cryostat (Leica RM2155, Nussloch, Germany).

### 2.5. Evans Blue Injections

To assess blood–brain barrier permeability, Evans blue (50 mg/kg, Sigma-Aldrich, St. Louis, MO, USA and Burlington, VT, USA) was intravenously injected through the tail vein and allowed to circulate in the rats for 4 h. Subsequently, rats were transcardially flushed with ice-cold saline followed by perfusion with PFA. Brain sections were then cut and imaged to evaluate permeability.

### 2.6. The Culture of bEnd.3 Cells and Oxygen–Glucose Deprivation (OGD) Exposure

Dulbecco’s modified eagle medium/F12 (DMEM/F12, 11320033, Gibco, Waltham, MA, USA) supplemented with 10% fetal bovine serum (FBS, 6000044, Gibco, USA) and 1% penicillin–streptomycin (15070063, Gibco, USA) was used to culture the mouse brain endothelial cell line, bEnd.3. The medium was changed every two days. Cells were seeded on a 6-well plate or 24-well plate at a density of 5 × 10^4^ cells/cm^2^. Sterile glass slides were placed in 24-well cell culture plates. The cells were washed twice with PBS, and the medium was replaced with either glucose-free DMEM (11966025, Gibco, USA) for OGD, which represents a common in vitro model of ischemic stroke used extensively in basic and preclinical stroke research [22,23], or DMEM/F12 (11320033, Gibco, USA) for control treatments, respectively. The cell plates in the OGD group were transferred to a hypoxia chamber (Billups-Rothenberg Inc., San Diego, CA, USA), and the air was replaced with OGD gas (95% N_2_ and 5% CO_2_). Cells were exposed to OGD conditions for 18 h at 37 °C [24]. For the mechanism of eNOS studies, bEnd.3 cells were pretreated with 400 μM NOS inhibitor L-NG-Nitro arginine methyl ester (L-NAME, 483125-M, Sigma, USA) [20] for 60 min before PBMT.

### 2.7. MTT Assay

The MTT cell viability assay (M6494, Thermo Fisher Scientific, Inc., Waltham, MA, USA) () was employed following the manufacturer’s instructions. The bEnd.3 cells were seeded at a density of 3 × 10^3^ cells/well into 96-well plates and cultured with different concentrations of L-NAME (ranging from 0 µM to 1000 mM) for 48 h at 37 °C, following a 4-hour incubation period with 20 µL of MTT solution per well. Subsequently, the absorbance of each sample was measured at a wavelength of 450 nm using a microplate reader (Thermo Fisher Scientific Inc., Waltham, MA, USA).

### 2.8. Immunofluorescence Staining and Microscopy

Immunofluorescence staining was conducted following procedures outlined in a previous study [17]. In brief, brain slices were prepared, and 3–5 sections from each animal were chosen for microscopy imaging. The collected coronal slices underwent permeabilization with 0.4% Triton X-100 for 8 h followed by blocking with 3% BSA for 1 h at room temperature. Subsequently, overnight incubation at room temperature was carried out using the respective primary antibodies. The following primary antibodies and reagents used included a senescence green detection kit (1:300, C10850, Invitrogen Corporation, Carlsbad, CA, USA), Reca1 (1:300, 14-0360-82, Invitrogen, Corporation, Carlsbad, CA, USA), endostatin (1:300, PA1-601, Thermo Fisher Scientific Inc., Waltham, MA, USA), CD31 (1:300, AF3628, R&D system, Minneapolis, MN, USA), P21 (1:300, SC-6246, Santa Cruz Biotechnology Inc., Dallas, TX, USA), p-eNOS (Ser1177, 1:300, MAB9028, Bio-techne, Minneapolis, MN, USA), and γH2AX (1:300, 80312s, Cell Signaling Technology Inc., Danvers, MA, USA). On the following day, brain slices were washed three times with 0.1% Triton X-100 and then incubated with Alexa Fluor-labeled anti-mouse/goat/rabbit secondary antibodies (647/488/568, Thermo Fisher Scientific, Inc., Waltham, MA, USA) for 1 h at room temperature. After washing, the sections were mounted with DAPI fluoromount-G^®^ mounting medium (SouthernBiotech, Birmingham, AL, USA).

For immunofluorescence staining of the cells, cells were cultured on coverslips, PBS-rinsed, and fixed with 4% PFA for 15 min. Subsequently, permeabilization was performed for 10 min using 0.4% Triton X-100 in PBS. Following this, coverslips were subjected to overnight incubation with the primary antibody at 4 °C. After three PBS washes, the secondary antibody was applied, and coverslips were incubated for 1 h at room temperature. Finally, the nuclei were stained with DAPI, and coverslips were mounted on glass slides and imaged.

All fluorescent images were captured using a Zeiss AxioObserver with ApoTome (Carl Zeiss, Maple Grove, MN, USA) or Olympus confocal microscope (Olympus Corporation, Tokyo, Japan) equipped with 20× or 40× oil immersion objective. Investigators used ImageJ 1.52a software (Java 1.8.0, Bethesda, MD, USA) to evaluate the images. Z-stack pictures were taken at intervals of 1 µm. Consistency was maintained across all imaging procedures with regard to exposure time and emission gain for all groups.

### 2.9. Automated Western Immunoblotting

As previously reported in our study [17], the Jess™ Simple Western system (ProteinSimple, San Jose, CA, USA) was used to assess protein expression of P21 (1:20, SC-6246, Santa Cruz Biotechnology Inc., Dallas, TX, USA) in cell samples. Samples were homogenized in tissue extraction reagent (FNN0071, Invitrogen Corporation, Carlsbad, CA, USA) containing protease and phosphatase inhibitors (78420, A32955, Thermo Scientific^TM^ Inc., Waltham, MA, USA). Subsequently, a Pierce BCA protein assay kit (23227, Thermo Scientific Inc., Waltham, MA, USA) was used to test the protein concentration of the lysates. The Simple Western system (ProteinSimple Ltd., San Jose, CA, USA) was used to perform capillary Western analysis according to the manufacturer’s instructions.

### 2.10. Statistical Analysis

During data acquisition and analysis, the investigators were blinded to treatment assignment. All data were tested for normality using the Shapiro–Wilk test and presented as mean ± standard deviation (SD). Statistical analysis was conducted using GraphPad Prism (Version 8.3.0, GraphPad, Boston, MA, USA). Comparisons between multiple groups were made using one-way ANOVA, with Tukey’s post hoc test. A significance level of *p* < 0.05 was deemed statistically significant.

## 3. Results

### 3.1. PBMT Reduced PT-Stroke-Induced Permeabilization of the BBB and Decreased Endostatin Levels in the Peri-Infarct Region

**Figure 2:**
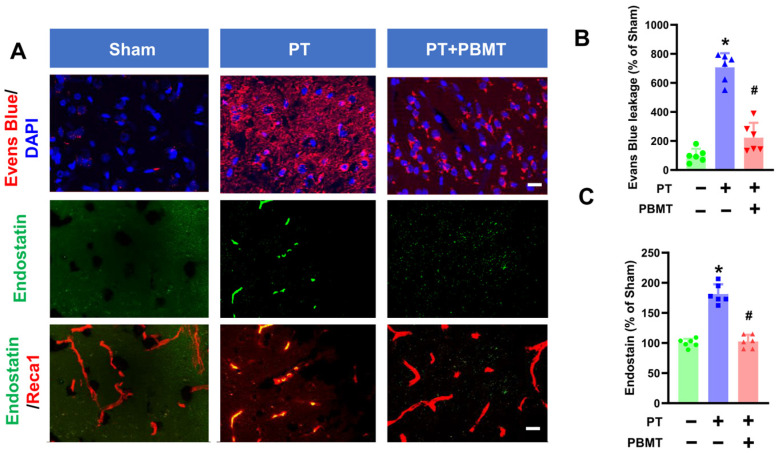
PBMT diminished the blood–brain barrier (BBB) permeability and decreased endostatin levels in PTstroke rats. (**A**) Representative fluorescence images of Evans blue (red), endostatin (green), and Reca1 (red) in the peri-infarct zone. Nuclei were counterstained with DAPI (blue). (**B**,**C**) Quantitative analysis of Evans blue leakage and vessel-associated endostatin was performed using ImageJ software (n = 5–6). The data were expressed as percentage changes versus the respective sham group. * indicates *p* < 0.05 vs. sham group; # indicates *p* < 0.05 vs. PT-stroke group. Scale bar = 20 μm.

Stroke causes the breakdown of the BBB leading to the influx of cytotoxic substances [25]. As depicted in Figure 2A,B, significant leakage of Evans blue was evident in the cortical peri-infarct area of PT animals (707.1 ± 97.67 v. 100.0 ± 46.6), which was substantially ameliorated in PBMT-treated rats (223.1 ± 102.0). In addition to BBB breakdown, neovascularization is prevalent following stroke and plays an essential role in repairing brain tissue. We further study the impact of PBMT on neovascularization by measuring endostatin levels in the peri-infarct region via immunohistochemistry. Endostatin has been reported to exhibit anti-angiogenic activity and plays as a negative regulator in neovascularization [26]. Co-labeling for Reca1, a vascular endothelial marker, revealed that endostatin levels were significantly elevated in the peri-infarct area of the PT stroke group (100.0 ± 6.96 v. 181.3 ± 16.27). Conversely, PBMT treatment attenuated this alteration (102.6 ± 11.03) (Figure 2C).

### 3.2. PBMT Inhibited Cerebrovascular Senescence in PT-Stroke Rats

**Figure 3:**
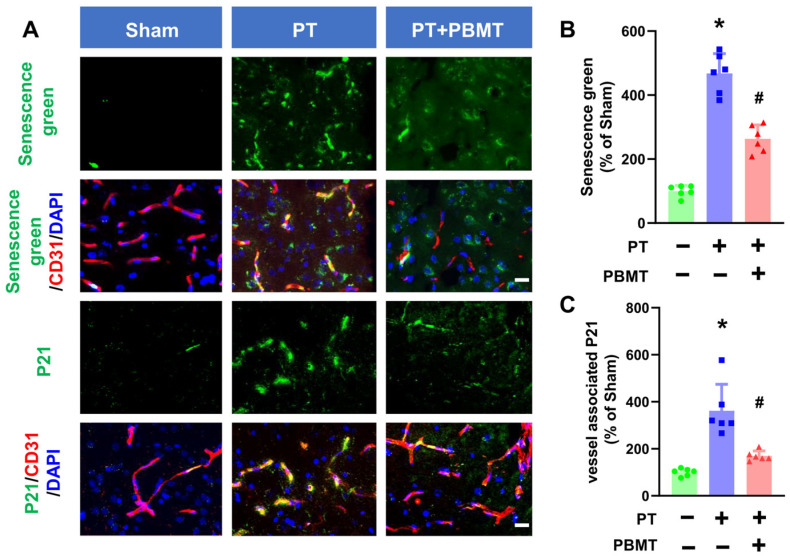
PBMT inhibited cerebrovascular senescence in PT-stroke rats. (**A**) Representative fluorescence images of senescence green (green), P21 (green), and CD31 (red) in the peri-infarct zone. Nuclei were counterstained with DAPI (blue). (**B**,**C**) Quantitative analysis of vessel-associated senescence green and P21 in the peri-infarct zone of rats. The data were expressed as percentage changes versus the respective Sham group. * indicates *p* < 0.05 vs. sham group; # indicates *p* < 0.05 vs. PT-stroke group. Scale bar = 20 μm.

Activation of SA-β-gal is commonly used as a marker of senescence. A fluorescein-based probe targeting SA-β-gal was utilized to detect cellular senescence following PT stroke in the presence/absence of PBMT (Figure 3A,B). Endothelial senescence was further confirmed by colocalization of senescence markers (P21) and vascular endothelial markers (CD31) in the peri-infarct region (Figure 3A,C). PT stroke led to a significant increase in vascular-associated senescence green and P21 in the peri-infarct region of the PT-stroke group (senescence green 361.55 ± 112.60; P21 467.73 ± 62.26), while animals treated with PBMT exhibited a dramatic decrease in vascular senescence (senescence green 169.09 ± 21.61; P21 263.50 ± 43.10).

### 3.3. PBMT Increased the Phosphorylation of eNOS at Ser1177 in Both PT-Stroke Rats and bEnd.3 Cells

**Figure 4:**
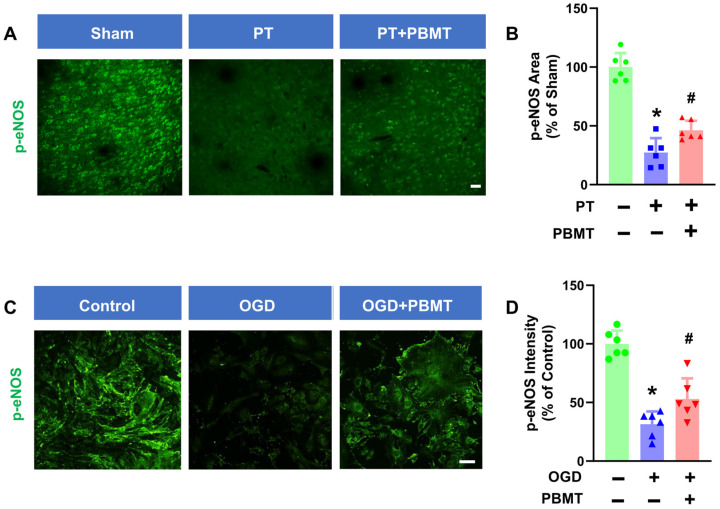
PBMT increased the phosphorylation of eNOS at Ser-1177 in PT-stroke rats and OGD-exposed bEnd.3 cells. (**A**) Representative fluorescence images of p-eNOS (green) in the peri-infarct zone. (**B**) Quantitative analysis of p-eNOS fluorescence intensity in the peri-infarct zone of rats. The data were expressed as percentage changes versus the respective sham group. Scale bar = 50 μm. (**C**) Representative immunofluorescence images for p-eNOS (green). (**D**) p-eNOS intensity was calculated and expressed as percentage changes relative to the control group. Scale bar = 50 µm (n = 6). * indicates *p* < 0.05 vs. sham or control group; # indicates *p* < 0.05 vs. PT-stroke or OGD group.

It has been well established that phosphorylation of eNOS at Ser1177 is a central regulator of endothelial function and homeostasis [27]. Phospho-eNOS antibody was further utilized to investigate whether 7-day PBMT could enhance eNOS phosphorylation (Figure 4A). The intensity of p-eNOS immunostaining was significantly reduced in PT-stroke rats as compared to the sham group (100.0 ± 12.0 vs. 27.32 ± 12.28), while treatment with PBMT increased expression of p-eNOS at Ser1177 (46.18 ± 8.12) (Figure 4B), indicating that PBMT is essential for eNOS activation in vivo.

To further confirm the effect of PBMT on eNOS activation in vitro, we assessed phospho-eNOS (Ser1177) levels in bEnd.3 cells. We discovered that exposure of bEnd.3 cells to OGD for 18 h led to a decrease in p-eNOS (100.0 ± 11.28 v. 31.47 ± 10.90). In contrast, PBMT + OGD significantly increased eNOS phosphorylation as compared to the OGD-only group (53 ± 17.52) (Figure 4C,D).

### 3.4. Dose-Dependent Cell Viability of bEnd.3 Cells Treated with L-NAME

**Figure 5:**
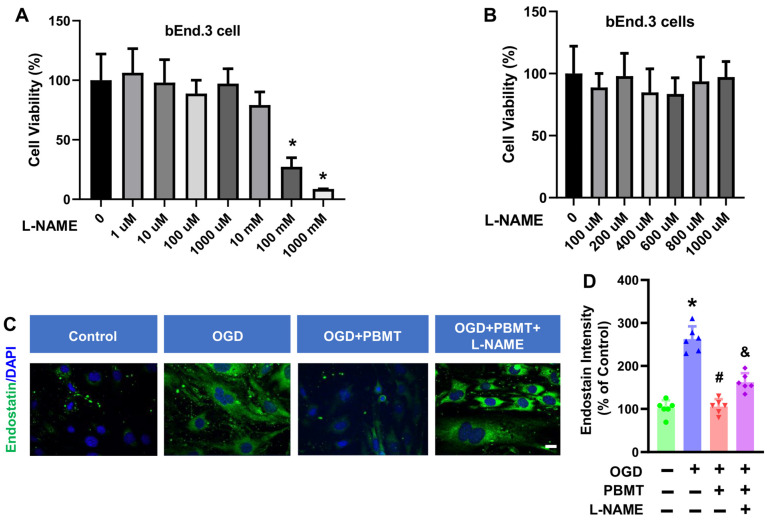
Effect of L-NAME on the cell viability and endostatin level in bEnd.3 cells. (**A**,**B**) MTT assay was used to measure viability in bEnd.3 cells after treatment with L-NAME at 1 μM to 1000 mM concentrations in bEnd.3 cells. The data are expressed as mean ± SD (n = 5–6). (**C**) Representative fluorescence images of endostatin (green) in the bEnd.3 cells. (**D**) Quantitative analysis of endostatin intensity. The data were expressed as percentage changes versus the respective control group. (n = 5–6). * indicates *p* < 0.05 vs. control group; # indicates *p* < 0.05 vs. OGD group. & indicates *p* < 0.05 vs. OGD + PBMT group. Scale bar = 20 μm.

We next determined whether eNOS mediates the beneficial effects of PBMT on endothelial functions in vitro. A NOS inhibitor, L-NAME, was used to inhibit eNOS activity in bEnd.3 cells. We tested 7 different doses of L-NAME (1 µM to 1000 mM) on bEnd.3 cell viability and observed that L-NAME had minimal impact on bEnd.3 cell viability until the addition of 100 mM L-NAME for 48 h (27.37 ± 7.56, Figure 5A,B). Therefore, we chose the 400 μM L-NAME as the intervention dose. This dose (400 μM) was also based on the previous publication suggesting that 400 μM L-NAME can reduce the NO production in vitro [20].

### 3.5. L-NAME Prevented PBMT-Inhibited Endostatin Level in the bEnd.3 Cell

In order to assess whether eNOS is the key element controlling the antiangiogenic factor, endostatin, following PBMT treatment, we performed immunostaining for endostatin in bEnd.3 cells. As shown in Figure 5C, an elevation of endostatin was observed following OGD (100.0 ± 18.45 v. 262.6 ± 29.76), but this was reversed by PBMT (105.6 ± 17.27). Interestingly, the PBMT-mediated inhibitory effect on endostatin production was abolished in the presence of L-NAME (162.3 ± 20.88, Figure 5D). Overall, these results highlight the involvement of eNOS in PBMT-mediated angiogenic effects.

### 3.6. L-NAME Hindered the Inhibitory Effect of PBMT on Endothelial Senescence

**Figure 6:**
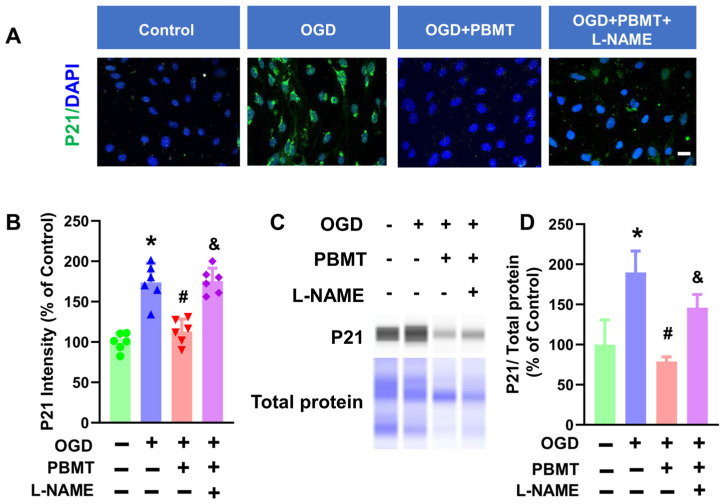
Pretreatment with L-NAME prevented PBMT-inhibited cellular senescence in bEnd.3 cells. (**A**) Representative fluorescence images of P21 (green) in the bEnd.3 cells. Nuclei were counterstained with DAPI (blue). (**B**) P21 staining intensity was analyzed and expressed as percentage changes versus the control group. (**C**,**D**) Western blotting and quantitative analysis of P21 levels using protein samples from bEnd.3 cells (n = 3–6). * indicates *p* < 0.05 vs. control group; # indicates *p* < 0.05 vs. OGD group. & indicates *p* < 0.05 vs. OGD + PBMT group. Scale bar = 20 μm.

The role of eNOS in PBMT-induced protection against vascular senescence in bEnd.3 cells was studied via immunocytochemistry. As shown in Figure 6A–C, PBMT significantly inhibited elevated P21 fluorescence intensity following OGD (173.8 ± 23.57 vs. 113.0 ± 15.47). This elevation of P21 was drastically reduced following eNOS inhibition via L-NAME (175.5 ± 16.03). An independent approach (e.g., Western blot analysis) was next performed to confirm the results obtained from the immunocytochemistry study. As expected, OGD-induced enhancement of P21 protein expression was suppressed following PBMT (100.0 ± 30.77 v. 190.0 ± 26.56). PBMT-mediated protection against endothelial senescence was abrogated in the presence of L-NAME (78.78 ± 5.84 vs. 145.90 ± 16.56), suggesting that eNOS is associated with the beneficial effect of PBMT in inhibiting vascular senescence.

### 3.7. Inhibition of DNA Damage by PBMT in bEnd.3 Cells Was Eliminated in the Presence of L-NAME

**Figure 7:**
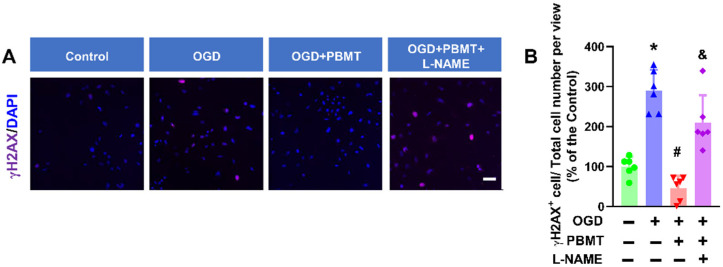
Pretreatment with L-NAME prevented a PBMT-induced decrease in histone H2AX phosphorylation in bEnd.3 cells. (**A**) Representative immunofluorescence images for γH2AX (purple). Nuclei were counterstained with DAPI (blue). (**B**) The ratio of γH2AX+ cell and total cell numbers was calculated and expressed as percentage changes relative to the control group. Scale bar = 50 µm (n = 6). * indicates *p* < 0.05 vs. control group; # indicates *p* < 0.05 vs. OGD group. & indicates *p* < 0.05 vs. OGD + PBMT group.

Cellular senescence is induced in response to DNA damage in vascular cells [28,29]. γH2AX is the most widely used marker of DNA damage [30]. We found that OGD significantly increased γH2AX+ cells (289.80 ± 52.22 vs. 100.0 ± 24.05), while PBMT treatment reduced DNA damage following OGD (45.64 ± 30.77). The beneficial effect of PBMT in inhibiting OGD-induced DNA damage was diminished in bEnd.3 cells subjected to L-NAME (209.9 ± 68.66, Figure 7A,B).

## 4. Discussion

Endothelial cells, lining the interior of blood vessels, are one of the components of the blood–brain barrier (BBB) and play a crucial role in regulating vascular function [31,32]. Endothelial senescence, which is associated with compromised BBB integrity [33], is implicated in the pathogenesis of several neurodegenerative disorders, including Alzheimer’s disease [34], stroke [35], and vascular dementia [36]. Our study aligns with previous research suggesting that vascular-associated senescence markers, including P21 and senescence green, were upregulated at 7 days post-PT-stroke. PBMT has been recognized for its anti-aging effects in peripheral tissues. PBMT can mitigate age-related cardiovascular remodeling, improve cardiac function, and enhance neuromuscular coordination [36]. We revealed in the present study that PBMT effectively reduced the cerebrovascular senescence and BBB dysfunction induced by stroke.

In addition to BBB integrity, angiogenesis plays a vital role in recovering neurological functions after stroke [37,38]. Endostatin is a well-studied endogenous angiogenesis inhibitor that exhibits potent antiangiogenic activity [39]. Clinical studies have highlighted plasma endostatin as a prognostic marker for cognition impairment during the acute phase of stroke [40,41,42]. Consistent with these findings, our results suggest an elevation of vessel-associated endostatin levels seven days after PT stroke. Interestingly, PBMT treatment reversed it, which suggests PBMT promotes angiogenesis and enhances cerebrovascular function.

Increasing evidence suggests that vascular NO production through eNOS is pivotal for vascular integrity and homeostasis [27,43,44] during normal physiological conditions, as augmented eNOS activates telomerase to delay endothelial senescence [45,46]. Conversely, hypoxia impedes eNOS phosphorylation at Ser-1177 and aggravates pulmonary hypertension [47,48]. Consistent with these observations, our results from in vivo studies revealed that eNOS phosphorylation was decreased in the peri-infarct area following PT stroke, while this phenomenon was reversed in PT-stroke animals subjected to PBMT treatment.

Given that the observed alterations in eNOS phosphorylation coincide with changes in vascular function following stroke and PBMT, we hypothesize that NOS may play a pivotal role as a mediator of the beneficial effects induced by PBMT on cerebrovascular function post-ischemic-stroke. To validate this, we investigated the role of NOS in PBMT’s effects on endothelial function using the bEnd.3 cell line in vitro. Our results indicate that the reduction in endostatin levels by PBMT in bEnd.3 cells following OGD were attenuated by L-NAME, a NOS inhibitor, suggesting that eNOS is crucial for PBMT’s inhibitory effect on endostatin induction.

We were aware that L-NAME can inhibit inducible nitric oxide synthase (iNOS) in ECs. Nevertheless, L-NAME inhibits the proliferation and differentiation of ECs [49,50], which is contradictory to iNOS’s pro-angiogenic effect in this study. In addition, the specificity of L-NAME for inhibition of eNOS over other related NOS (e.g., iNOS) is higher than 10-fold [51]. In fact, L-NAME has been widely employed to inhibit eNOS activity in various cellular studies [52,53,54]. Although it has been reported that iNOS expression and cellular death can be observed in endothelial cells (ECs) 4 h after OGD [55], iNOS levels return to normal levels 25 h after OGD [51,56]. We discovered in the present study that endostatin levels were increased by L-NAME at 48 h following OGD, suggesting that L-NAME modulates endostatin in an iNOS-independent manner. DNA damage is prevalent following stroke, which leads to eNOS uncoupling. Instead of NO, uncoupled eNOS produces a superoxide anion serving as a harmful free radical to exacerbate oxidative stress and DNA damage [44,57]. We are the first to report that PBMT at a dose of 3 J/cm^2^ reduced DNA damage levels, evidenced by diminished γ-H2AX expression in endothelial cells.

The mechanism underlying PBMT-mediated eNOS phosphorylation and DNA protection in ECs remains largely unknown. PBMT has been reported to activate several calcium ion channels (such as transient receptor potential channels), thereby increasing cytosolic calcium concentration [58,59,60]. It is important to note that eNOS activity is contingent upon calmodulin and is subject to physiological regulation by intracellular Ca^2+^ concentration. DNA protection mediated by PBMT was also inhibited in the presence of L-NAME, underscoring that eNOS is a key mediator of PBMT-induced DNA protection. eNOS phosphorylation at Ser-1177 and NO photodissociation from cytochrome c oxidase (COX) complex IV in mitochondria [61] can inhibit ROS-related mitochondrial damage, thus reducing DNA damage and preventing endothelial senescence [62,63,64].

## 5. Conclusions

**Figure 8:**
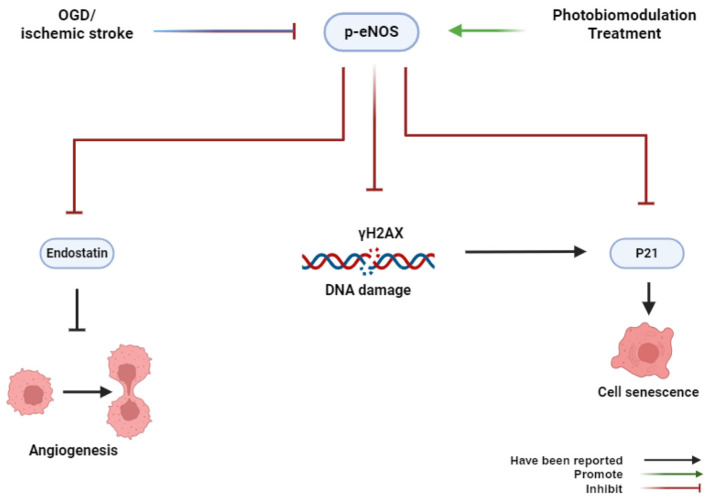
Graphical abstract. OGD or ischemic stroke inhibits the phosphorylation of eNOS, induces histone H2AX phosphorylation, and causes cerebrovascular senescence. Conversely, PBMT promotes the phosphorylation of eNOS, induces cell proliferation, and inhibits cerebrovascular senescence.

In summary, our findings, for the first time, provide novel insights into the role of PBMT in preventing cerebrovascular senescence in the peri-infarct microvasculature through eNOS phosphorylation and anti-DNA damage (Figure 8). Our study suggests a new therapeutic approach to alleviate endothelial dysfunction caused by cerebrovascular disease.

## Data Availability

The data presented in this study are available on request from the corresponding author.
