# Correction: Feng et al. Photobiomodulation Inhibits Ischemia-Induced Brain Endothelial Senescence via Endothelial Nitric Oxide Synthase. *Antioxidants* 2024, *13*, 633

**DOI:** 10.3390/antiox13081005

**Published:** 2024-08-19

**Authors:** Yu Feng, Zhihai Huang, Xiaohui Ma, Xuemei Zong, Vesna Tesic, Baojin Ding, Celeste Yin-Chieh Wu, Reggie Hui-Chao Lee, Quanguang Zhang

**Affiliations:** 1 Institute for Cerebrovascular and Neuroregeneration Research, Shreveport, LA 71103, USA; 2 Department of Neurology, Louisiana State University Health, Shreveport, LA 71103, USA; 3 Department of Biochemistry & Molecular Biology, Louisiana State University Health, Shreveport, LA 71103, USA

In the original publication [1], there was a mistake in Figure 7A as published. It showed incorrect representative immunofluorescence images. The corrected Figure 7A is provided below. The authors state that the scientific conclusions are unaffected. This correction was approved by the Academic Editor. The original publication has also been updated. 

## Figures and Tables

**Figure 7:**
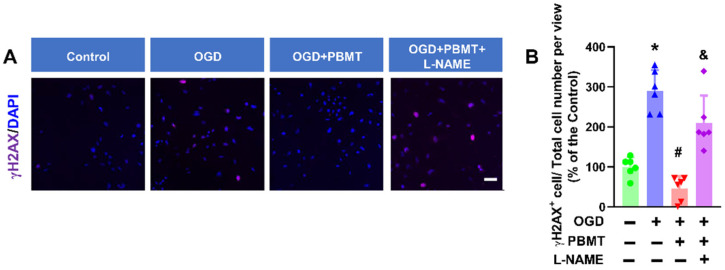
Pretreatment with L-NAME prevented a PBMT-induced decrease in histone H2AX phosphorylation in bEnd.3 cells. (**A**) Representative immunofluorescence images for γH2AX (purple). Nuclei were counterstained with DAPI (blue). (**B**) The ratio of γH2AX+ cell and total cell numbers was calculated and expressed as percentage changes relative to the control group. Scale bar = 50 µm (n = 6). * indicates *p* < 0.05 vs. control group; # indicates *p* < 0.05 vs. OGD group. & indicates *p* < 0.05 vs. OGD + PBMT group.
